# Selective Dry-Off Therapy in Conventional Dairy Farms: The Influence of Quarter-Level Selection Criteria on Postpartum Mastitis and Somatic Cell Count

**DOI:** 10.3390/ani15213167

**Published:** 2025-10-31

**Authors:** Jaromír Ducháček, Veronika Legarová, Radim Codl, Lucie Kejdová Rysová, Matúš Gašparík, Soňa Formánková Herman, Hana Nejeschlebová

**Affiliations:** 1 Department of Animal Science, Faculty of Agrobiology, Food and Natural Resources, Czech University of Life Sciences Prague, Kamýcká 129, 165 00 Prague, Czech Republic; duchacek@af.czu.cz (J.D.); codl@af.cuz.cz (R.C.); gasparikm@af.czu.cz (M.G.); 2 Department of Food Science, Faculty of Agrobiology, Food and Natural Resources, Czech University of Life Sciences Prague, Kamýcká 129, 165 00 Prague, Czech Republic; rysoval@af.czu.cz (L.K.R.); hermanovas@af.czu.cz (S.F.H.); 3 Dairy Research Institute Ltd., Ke Dvoru 12a, 6-Vokovice, 160 00 Prague, Czech Republic; hana.nejeschlebova@seznam.cz

**Keywords:** selective dry cow therapy, somatic cell count, mastitis, antibiotic reduction, udder health

## Abstract

**Simple Summary:**

This study evaluated the use of selective dry cow therapy in two commercial dairy farms in the Czech Republic to reduce antibiotic use without harming udder health or milk quality. Milk quarter samples were collected before and after drying-off to analyze somatic cell counts, microbial infections, and milk composition. The results showed no significant differences in udder health between quarters treated with or without antibiotics when appropriate selection criteria were applied. However, quarters treated with antibiotics tended to have a higher clinical mastitis incidence, likely due to pre-existing infections. These findings support the use of selective dry cow therapy as a sustainable approach to lower antibiotic use in dairy farming while maintaining animal welfare and milk quality. Improved diagnostic methods and careful herd management are essential for successful implementation.

**Abstract:**

The present study assessed the efficacy of selective dry cow therapy (SDCT) on two commercial Holstein-Friesian farms in the Czech Republic, involving 572 quarter milk samples from 74 cows collected over a two-year period. Quarter samples were taken both at dry-off (n = 296) and post-calving (n = 276) to assess somatic cell count (SCC), cultured microbial results (counts), milk composition, and mastitis incidence. The average SCC at dry-off was 264,000 cells/mL (SD = 650,000) in Farm 1 and 224,000 cells/mL in Farm 2. Mastitis incidence averaged 24.42% and 18.75% in Farms 1 and 2, respectively. Correlation analysis revealed significant associations between pre-dry-off milk parameters and post-calving udder health indicators, including negative correlations between SCC prior to drying-off and lactose content after calving (r = −0.161, *p* < 0.01). Statistical analyses showed a significant farm effect on cultured microbial occurrence and mastitis occurrence after calving (*p* < 0.05), as well as a significant influence of lactation number on postpartum mastitis and SCC (*p* < 0.05). Also, mastitis incidence was significantly higher (9.43%, *p* < 0.05) in treated quarters. The use of selective non-antibiotic dry cow therapy does not impair udder health and milk quality but helps reduce the risk of antibiotic resistance. Further refinement of diagnostic criteria is needed to optimize treatment decisions and improve herd-level outcomes.

## 1. Introduction

The dairy industry currently faces a critical challenge: how to effectively reduce the use of antibiotics while maintaining the health and productivity of dairy cattle. Antibiotics have traditionally played a central role in preventing and treating infections in livestock, particularly in intensive farming systems. However, the overuse and misuse of antimicrobial agents in animal agriculture significantly contribute to the global threat of antimicrobial resistance (AMR), which compromises the efficacy of these drugs in both veterinary and human medicine [1,2,3,4].

This is further exacerbated by the widespread prophylactic and metaphylactic use of antibiotics in livestock, including during the dry period in dairy cattle, when antibiotics are often administered to prevent mastitis rather than to treat an existing infection [5].

In response to this escalating health risk, the European Union has taken decisive legislative action aimed at reducing the routine use of antibiotics in animal husbandry. The implementation of Regulation (EU) 2019/6 on veterinary medicinal products and Regulation (EU) 2019/4 on medicated feed reflects a paradigm shift towards more sustainable and responsible use of antimicrobials in agriculture [6]. These 2022 regulations ban preventive antibiotic use in animal groups and restrict their application in non-clinical cases, such as routine dry-off in dairy cows [7].

This regulatory change encourages the adoption of alternative, non-antibiotic-based management strategies for udder health and disease prevention, including selective dry cow therapy (SDCT), enhanced hygiene practices, vaccination, and the use of internal teat sealants. Evidence from recent studies supports the efficacy of SDCT in reducing antibiotic usage without compromising animal welfare or milk quality, particularly when combined with regular somatic cell count (SCC) monitoring and pathogen-specific diagnostics [8,9,10].

Furthermore, the refinement of SDCT protocols towards quarter-level application—rather than traditional cow-level approaches—has shown additional potential to optimize antibiotic use by targeting only infected quarters while leaving healthy ones untreated [11]. Studies indicate that quarter-level SDCT, supported by culture-based or molecular diagnostic testing before drying-off, can achieve similar cure rates and prevent new infections as effectively as blanket dry cow therapy (BDCT), provided that robust hygiene and monitoring systems are in place [12]. This precision-based strategy aligns with the principles of prudent antimicrobial use and sustainable dairy management, reflecting the growing emphasis on evidence-based, pathogen-specific interventions to control intramammary infections at the end of lactation.

The dry period represents a critical phase in the lactation cycle of dairy cattle, during which involutional and regenerative processes occur within the mammary gland in preparation for the subsequent lactation. Traditionally, blanket dry cow therapy (BDCT) has been applied, involving the intramammary administration of antibiotics into all four quarters of all cows at drying-off, with the aim of both preventing new intramammary infections (IMI) and treating existing subclinical mastitis. However, this approach contributes significantly to the overuse of antibiotics, as most cows and quarters are not infected at dry-off, which further increases the development of antimicrobial resistance [9,13,14].

As a result, there is growing interest in SDCT, which involves the targeted use of antibiotics only in cows or udder quarters identified as being at high risk of infection [15]. SDCT relies on the identification of cows or quarters that are likely to benefit from antimicrobial treatment, typically based on a combination of criteria, including SCC, results of bacteriological culture, and records of previous clinical mastitis episodes [10,16,17].

SCC is widely recognized as a reliable indicator of udder health and milk quality, with elevated SCC values generally reflecting the presence of an intramammary infection [18]. Within the European Union, regulatory standards have established a legal limit for SCC in raw cow’s milk at 400,000 cells per milliliter, measured as a rolling geometric mean over a three-month period [19].

Although SDCT offers several advantages, its implementation faces multiple challenges. These include the need for accurate and reliable diagnostic tools, the development of effective selection criteria, and the management of udder health in the absence of routine antibiotic use. Furthermore, the success of SDCT depends on a range of factors, such as herd management practices, milking hygiene, and the prevalence and pathogenicity of mastitis-causing organisms [8,16,20]. The point of SDCT is to find out if the animals are healthy or infected at dry-off. There is a need for a broad definition of an infected cow or quarter to encompass different types of herd systems, considering not only an increase in somatic cell count [21], but also changes in electrical conductivity [22], a decrease in lactose [21], an increase in milk temperature [23], etc.

The hypothesis of this study is that, with appropriate drying-off recommendations, there will be no difference in SCC or clinical mastitis incidence after the dry period between antibiotic-treated and non-antibiotic-treated quarters at drying-off. The primary aim of this study is therefore to evaluate the efficacy of selectively drying-off quarters with antimicrobials in reducing antibiotic use while maintaining udder health and milk quality in dairy cattle.

By examining the outcomes of SDCT in dairy cows across different farms or herds and management systems, this research seeks to contribute to the development of best practices for the sustainable use of antibiotics in the dairy sector. The findings are intended to support evidence-based guidelines that promote responsible antimicrobial stewardship without compromising animal welfare or production efficiency.

## 2. Materials and Methods

The study was conducted in accordance with the Czech legislation for the protection of animals against abuse (no. 246/1992) [24] and with the directive 2010/63/EU [25] on the protection of animals used for scientific purposes. The experimental protocol was approved by the scientific board of the Czech University of Life Sciences.

In total, 572 quarter milk samples were collected and analyzed from 74 Holstein-Friesian cows (31 cows before dry-off in the 1st lactation; 25 cows in the second lactation and 18 in the third and subsequent lactations) housed on two commercial dairy farms located in the Czech Republic (Farm 1: n = 124 total quarter samples; Farm 2: n = 448 total quarter samples) between February 2023 and April 2024. All samplings were performed by trained study staff. Milk sampling was conducted at two specific time points. The first time point was prior to dry-off, when 296 quarters were assessed (Farm 1: n = 72 quarters; Farm 2: n = 224 quarters). Out of a total of 296 quarters on Farm 1 and Farm 2, 168 quarters were antibiotic-treated and 128 were non-antibiotic-treated. The second time point was in early lactation after calving (n = 276; Farm 1: n = 52 quarters; Farm 2: n = 224 quarters). Milk samples were collected individually from each quarter and also evaluated separately.

The distribution of cows by lactation number reflected the herd demographics. Some cows enrolled at the dry-off stage were culled during the dry period or shortly after calving due to postpartum health issues (n = 5).

Both farms were located within 50 km of Prague, in the Central Bohemian Region, at an elevation of 319 (Farm 1), respectively, 412 m (Farm 2) above sea level. Farm 1 maintained a herd of approximately 380 lactating cows, while Farm 2 managed around 120 cows. The cows were kept in free-stall barns with straw or recycled dried manure solids bedding. Cows were milked twice daily in a 2 × 12 side-by-side parlor (Farm 1) and a 2 × 12 herringbone parlor with rapid exit (Farm 2). Animals were included in the study regardless of their parity, mastitis history, or previous milk yield.

The first milk samples were collected approximately one week prior to drying-off. Postpartum samples (excluding those from culled or non-calved cows) were collected between days 6 and 21 of the subsequent lactation, as this is the critical period for the development of intramammary infections. The average dry period length was approximately 60 days for all quarters of all cows.

In addition to milk sampling, udder quality parameters, skin condition (hyperkeratosis, cleanliness), and zootechnical records were collected for each cow included in the study. All these parameters were evaluated and recorded by trained study staff. Furthermore, data about daily milk yield [kg], milk conductivity [mS], and blood content in milk [%] were measured by AfiLab milk analyzers (Afimilk, Afikim, Israel). Additional data about SCC were taken from milk performance control records, which are routinely performed for each cow once a month; however, these data were only used for one of the selection criteria for SDCT and are not presented in the results. Before sampling, the entire teat surface, including the teat end, was thoroughly cleaned using paper towels soaked in 70% ethanol. The first 3–4 streams of milk were discarded to minimize contamination from teat canal flora. The sampling protocol followed the official milk performance testing methodology used in the Czech Republic. Approximately 100 mL of milk was collected from each quarter on the farm into 50 mL sterile Falcon tubes (VWR International, Radnor, PA, USA).

All samples were labeled with the cow’s identification number, lactation number, and the specific quarter sampled. Samples were transported to the Milk Laboratory at the Czech University of Life Sciences Prague under refrigerated conditions (<6 °C) and processed promptly upon arrival.

Raw milk samples were homogenized using an IKA MS 3 vortex mixer (Fisher Scientific, Delft, The Netherlands) and subsequently warmed to 40 °C in a water bath. The major physicochemical parameters of milk—fat, protein, casein, lactose, total solids (TS), solids-not-fat (SNF), and freezing point (FP)—were measured using a MilkoScan FT 120 infrared analyzer (FossElectric, Hillerød, Denmark). The device was regularly calibrated in accordance with the manufacturer’s instructions to ensure accurate analysis of raw cow milk components.

Titratable acidity of the milk samples was determined by titration with a 0.25 M NaOH standard solution using phenolphthalein as an indicator. A 10 mL aliquot of each milk sample was titrated to the equivalence point, and the results were expressed in Soxhlet–Henkel degrees (°SH).

SCC in milk samples was analyzed using a Lactoscan SCC counter (Milkotronic Ltd., Nova Zagora, Bulgaria), which employs fluorescence microscopy for cell quantification. Approximately 100 µL of pre-warmed, homogenized milk was mixed with Sofia Green fluorescent dye in a microtube. The stained sample was homogenized, and 8 µL was applied into one chamber of a four-chamber disposable cartridge (Lachtochip 4R 50 µm 4 × 16, Milkotronic Ltd.). This procedure was repeated for each of the four quarters of every cow. Results were expressed in somatic cells per milliliter (cells/mL).

A work-based assessment scale was used to assess teat skin condition (Score 1: no wrinkling of the teat; Score 2: approximately 25% wrinkling of the teat surface; Score 3: moderate wrinkling, covering about 30–60% of the teat surface; Score 4: pronounced wrinkling, covering 60–80% of the teat surface; Score 5: severe wrinkling, 100% of the teat surface affected) [26]. The level of hyperkeratosis was assessed using the “Teat Condition Score” methodology (Score 1: no ring; Score 2: smooth or slightly rough ring; Score 3: rough ring; Score 4: very rough ring) [27]. Udder and teat cleanliness was assessed according to the methodology (Score 1: free of dirt; Score 2: slightly dirty, 2–10% of surface area; Score 3: moderately covered with dirt, 10–30% of surface area; Score 4: covered with caked-on dirt > 30% of surface area) [28].

Based on the evaluation of milk sample results and associated diagnostic parameters, recommendations for either antibiotic or non-antibiotic dry-off therapy were formulated for individual quarters. The decision-making process followed primary and secondary selection criteria, as outlined in Table 1 and Table 2. Evaluated parameters included: quarter-level SCC prior to dry off (the cut-off value of 100 × 10^3^/mL was chosen based on the recommendation of a veterinarian); microorganisms prior to dry off (quarter level); last milk production per day (cow level); clinical mastitis (cow level); titration acidity (quarter level); teat condition (quarter level); lactose content (quarter level); conductivity (cow level); blood content in milk (cow level); SCC from milk performance control (cow level); and occurrence of other diseases (cow level). Microbiological findings (microorganisms prior to dry off) were determined using cultivation on CM (ClearMilk) test (cultivation with a sterile microbiological loop under strict hygienic conditions, 24 h cultivation at 38.5 °C on chromogenic selective agar; LabMediaServis, Jaromer, Czech Republic) scored on a semiquantitative scale (none = 0, low (up to 6 colonies) = 20, moderate (from 7 to 10 colonies) = 50, high (up to 10 colonies) = 100). The ClearMilk test is a Petri dish with three parts of chromogenic selective agar: *Streptococcus*, *Staphylococcus*, and G^−^. CM test provides the ability to identify specific pathogens. However, in our study, we monitored the number of colonies grown and did not distinguish between specific major and minor pathogens. All positive findings were considered equivalent for selection decisions. None of our CM tests identified more than three pathogens in levels indicative of contamination. Clinical mastitis incidence was assessed as a percentage (0 = no mastitis, 100 = mastitis occurrence). Clinical mastitis cases were confirmed and then registered by the veterinarian. Cows were classified as clinically mastitic when they exhibited visible changes in milk (presence of blood, flakes, or clots) and alterations in physiological parameters (increased quarter temperature, redness, and heightened sensitivity to touch). For non-antibiotic dry-off of quarters, all main criteria and most secondary ones at the quarter level must be met, as well as for cows, as described above.

Statistical analyses were performed using SAS software version 9.4 (SAS/STAT^®^; SAS Institute, Inc., Cary, NC, USA). The GLM procedure was used for statistical evaluation. The best-fitting model was selected using the STEPWISE method in the REG procedure based on Akaike’s Information Criterion. Fixed effects included in the model equation were farm, recommendation to dry cow therapy, and linear regression on lactation order by dry cow therapy application (LOD). A detailed evaluation was conducted using Tukey’s test. The model equation was as follows:

Y_ijk_ = µ + FAi + REj + b*(LOD) + e_ijk_

where

- Y_ijk_ = measured value of the dependent variables (cultured microbial occurrence [%], occurrence of mastitis in udder quarters [%], SCC [×10^3^/mL]);
- µ = mean value of the dependent variable;
- FAi = fixed effect of farm (i = 1, n = 72; i = 2, n = 224);
- REj = fixed effect of recommendation to dry cow therapy (j = ATB (with using antibiotic), n = 168; j = NATB (without using antibiotic), n = 128);
- b*(LOD) = linear regression on lactation order by dry cow therapy application;
- e_ijk_ = residual errors.

Statistical significance levels used were *p* < 0.05, *p* < 0.01, and *p* < 0.001.

## 3. Results

Farm 1 had an average SCC at dry-off of 264,000 cells/mL with a standard deviation of 650,000 cells/mL. The average incidence of mastitis on this farm was 24.42%, and the mean microbial culture score at dry-off was 18.33 with a standard deviation of 30.20. The average daily milk yield on the sampling day prior to dry-off was 20.56 L with a standard deviation of 9.16 L.

Farm 2 was characterized by an average SCC of 224,000 cells/mL at dry-off, with individual values ranging from 4000 to 4,202,000 cells/mL. Mastitis incidence in this herd was 18.75%. The microbial culture score reached an average of 36.25 with a standard deviation of 36.61 at dry-off. The mean daily milk yield on the day of sampling before dry-off was 28.38 L, with a standard deviation of 5.46 L.

Cows in their first lactation had an average somatic cell count (SCC) of 97 × 10^3^ cells/mL prior to drying-off, with a standard deviation of 238 × 10^3^ cells/mL. Cows in their second lactation had an average SCC of 209 × 10^3^ cells/mL (SD = 470 × 10^3^ cells/mL) prior to drying-off. Cows in their third and later lactations had an average SCC of 567 × 10^3^ cells/mL (SD = 1059 × 10^3^ cells/mL) prior to drying-off.

Correlation analysis of the relationships between potential indicators of milk quality and mastitis occurrence before dry-off, and milk quality and mastitis-related parameters after calving, revealed several interesting associations. The most relevant correlations are summarized in Table 3. The cultured microbial occurrence before dry-off was negatively correlated with cultured microbial occurrence (r = 0.197, *p* < 0.01) after calving. The incidence of mastitis prior to dry-off was also correlated with post-calving lactose content (r = −0.148, *p* < 0.05).

Titratable acidity prior to dry-off was significantly correlated with lactose content (r = 0.289, *p* < 0.01) and teat skin condition (r = −0.184, *p* < 0.01). SCC before dry-off was associated with post-calving lactose content (r = −0.161, *p* < 0.05), and both teat skin condition and hyperkeratosis (r = 0.185 and 0.139, *p* < 0.01–0.05). Tendencies were also observed between SCC prior to drying-off and the occurrence of mastitis, as well as the somatic cell count after calving.

Furthermore, the content of basic milk components before dry-off (fat %, protein %, and lactose %) showed significant correlations with the corresponding post-calving milk component levels (r = −0.292 to 0.323, *p* < 0.01–0.05).

The detailed statistical analysis demonstrated a significant effect of farm on both microbial culture results and mastitis incidence (*p* < 0.05). Additionally, lactation number was identified as a significant predictor for postpartum mastitis occurrence and SCC in milk (*p* < 0.05).

Analysis of microbial culture outcomes based on model effects revealed a statistically significant difference between farms (*p* < 0.01; see Table 4).

Mastitis incidence data derived from herd records showed no significant differences between the two farms studied. Conversely, a significantly higher mastitis incidence (+9.43%, *p* < 0.05) was found in quarters subjected to antibiotic dry-off therapy compared to non-antibiotic-treated quarters.

SCC values after calving were relatively high across both farms (208.98 resp. 284.85 × 10^3^/mL), with a considerable variability indicated by large standard errors.

Non-antibiotic-treated quarters exhibited a notably higher mean SCC (310.210 × 10^3^/mL) alongside a high standard error, reflecting wide dispersion within this group. Overall, no significant differences were identified between antibiotic- and non-antibiotic-treated quarters in terms of dry-off recommendations, suggesting comparable outcomes based on the applied selection criteria.

## 4. Discussion

This pilot study investigated the effectiveness of SDCT in two commercial Holstein-Friesian farms in the Czech Republic, focusing on milk quality parameters, mastitis incidence, and the potential for reducing antibiotic use at the quarter level. The two farms differed markedly in baseline udder health indicators. At dry-off, the first herd exhibited a mean SCC of 264 × 10^3^ cells/mL, whereas the second herd averaged 224 × 10^3^ cells/mL with extreme variability. Mastitis incidence was also numerically higher in Farm 1 (24.42%) compared to Farm 2 (18.75%), while the microbial culture score at dry-off was twice as high in Farm 2 (36.25% vs. 18.33%), indicating a greater pathogen load despite lower SCC. These results correspond to the average values determined as part of the milk performance control.

Expectedly, SCC at dry-off increased with lactation number, supporting the established link between parity and intramammary infection susceptibility. In line with this, lactation number significantly predicted both postpartum SCC and mastitis occurrence [15].

Correlation analysis revealed biologically meaningful links between pre-dry-off indicators and post-calving outcomes. Higher SCC before dry-off was associated with lower lactose postpartum (r = −0.161) and poorer teat condition (r = 0.185), while cultured microbial occurrence prior to dry-off correlated with hyperkeratosis postpartum (r = 0.197). Mastitis incidence before dry-off negatively affected post-calving lactose (r = −0.148), indicating carry-over effects on milk composition. Moreover, fat, protein, and lactose contents before dry-off strongly correlated with their post-calving levels (r = −0.292 to 0.323), reinforcing the predictive utility of late-lactation milk profiling for early lactation milk quality. These results highlight the connection between milk quality parameters and udder tissue health, and mastitis infection [5]. These findings emphasize the value of integrating multiple diagnostic criteria—such as SCC, microbiological testing, and clinical observations—in developing robust selection protocols for SDCT.

Our study confirmed a significant farm effect on microbial culture results, highlighting that mastitis incidence is influenced by herd-specific management practices and environmental factors affecting udder health. These findings are consistent with those reported in several previous studies [20,29,30]. Our study’s confirmation of lactation number as a predictor of postpartum mastitis and SCC aligns with established knowledge that older cows are generally more susceptible to intramammary infections [10].

The lack of significant differences in microbial culture results between antibiotic- and non-antibiotic treated quarters within farms supports the potential of SDCT to maintain udder health without routine blanket antibiotic use. This finding is in agreement with studies demonstrating that targeted antibiotic therapy based on SCC and microbial diagnostics can reduce antimicrobial usage without compromising health outcomes [8,9,31].

One of the most striking and practically relevant findings of this study was that, despite no significant difference in mastitis incidence at the herd level between farms, quarters treated with antibiotics at dry-off showed a markedly higher mastitis occurrence (+9.43%) compared to non-antibiotic-treated quarters. This seemingly paradoxical outcome strongly suggests that antibiotic-treated quarters already represented a high-risk or subclinically infected population prior to drying-off, highlighting the critical importance of accurate and evidence-based selection criteria to ensure that antibiotic therapy is targeted only where truly necessary and not applied routinely [15,32,33].

Relatively high SCC values with large variability were recorded in both farms, particularly in non-antibiotic-treated quarters. This variability suggests a heterogeneous response among animals and quarters, which may be influenced by factors such as pathogen type, individual immune response, and management conditions. Nevertheless, the absence of significant differences in dry-off recommendations between antibiotic- and non-antibiotic groups indicates that SDCT protocols, when properly implemented, can effectively balance udder health preservation and antibiotic reduction goals.

Overall, this research contributes to the growing body of evidence supporting SDCT as a sustainable strategy for reducing antimicrobial use in dairy production without compromising animal welfare or milk quality. Our results demonstrate the importance of evaluating the criteria for selectively drying-off cows based on quarter-level information. Introducing new criteria at the quarter and cow level and setting strict limits can reduce the overall incidence of mastitis and help select animals for non-antibiotic dry-off.

Future work should focus on refining diagnostic thresholds, exploring herd-specific risk factors, and evaluating long-term impacts of SDCT on udder health and production efficiency.

## 5. Conclusions

This study demonstrated that SDCT can be effectively implemented in commercial dairy herds without compromising udder health or milk quality. Significant differences between farms in microbial culture results and mastitis incidence highlight the influence of herd-specific management practices.

Correlation analyses underscored the value of integrating multiple diagnostic indicators, including SCC, cultured microbial occurrence, and milk composition parameters, to optimize treatment decisions. Overall, the findings contribute valuable evidence towards sustainable antibiotic stewardship in dairy production.

No significant differences were observed between antibiotic- and non-antibiotic-treated quarters in terms of microbial culture outcomes or dry-off recommendations, supporting the potential of SDCT to reduce antibiotic use while maintaining health standards. However, antibiotic-treated quarters showed a higher mastitis incidence, likely reflecting pre-existing infection risk, emphasizing the need for accurate selection criteria. Infected cows, despite antibiotic treatment, are at risk of higher clinical mastitis incidence, indicating that other management decisions, such as culling, should be considered.

Overall, the shift towards reduced antibiotic use in dairy farming is not only a regulatory imperative but also a key component of global public health efforts to combat antimicrobial resistance. Successful implementation depends on a multidisciplinary approach involving veterinarians, farmers, policymakers, and researchers to promote best practices that ensure both animal health and sustainable milk production.

Results of this study should be further verified on more farms and animals across the EU. Future research should focus on refining diagnostic protocols and assessing the long-term effects of SDCT on herd health and productivity.

## Figures and Tables

**Table 1:** Main criteria for selective dry cow therapy.

Dry Cow Therapy	Main Criteria			
	Quarter-Level SCC Prior to Dry off	Microorganisms Prior to Dry off	Last Milk Production per Day	Clinical Mastitis BR
ATB	>100 × 10^3^/mL	moderate, high score	over 12 kg	occurrence in lactation
NATB	<100 × 10^3^/mL	none, low score	up to 12 kg	without occurrence in lactation

SCC—somatic cells count, ATB—antibiotic, NATB—non antibiotic, BR—breeding records.

**Table 2:** Secondary criteria for selective dry cow therapy.

Dry Cow Therapy	Secondary Criteria						
	Titration Acidity	Teat Condition	Lactose Content (%)	Conductivity	Blood Content in Milk	SCC from MPC	Occurrence of Other Diseases
ATB	<6 or >7.8 SH	>3 (skin condition, hyperkeratosis, cleanliness)	<4.9	unbalanced	over 0.3%	min. 1× MPC in lactation over 400 × 10^3^/mL	one month before with
NATB	6–7.8 SH	<3 (skin condition, hyperkeratosis, cleanliness)	>4.9	balanced	to 0.3%	all MPC in lactation under 400 × 10^3^/mL	one month before without

SCC—somatic cells count, ATB—antibiotic, NATB—non antibiotic, MPC—milk performance control.

**Table 3:** Correlation between selected milk quality and mastitis indicators prior to drying-off and after calving.

Prior to Drying-Off / After Calving		Mastitis in Udder Quarters (%)	TA (SH)	SCC (×10^3^/mL)	F (%)	P (%)	L (%)	Cultured Microbial Occurrence (%)	Teat Skin Condition	Udder Cleanliness	Hyperkeratosis Occurrence
Cultured microbial occurrence (%)	r	0.055	0.050	0.043	0.004	0.040	−0.100	0.197	0.112	0.103	−0.031
	*p*	0.367	0.415	0.479	0.954	0.514	0.100	<0.01	0.067	0.091	0.611
	n	272	270	271	236	270	270	239	271	271	271
Mastitis in udder quarters (%)	r	0.062	0.097	0.044	−0.050	−0.012	−0.148	−0.044	0.090	0.065	0.106
	*p*	0.310	0.111	0.472	0.449	0.848	0.015	0.502	0.141	0.286	0.081
	n	272	270	271	236	270	270	239	271	271	271
TA (SH)	r	−0.037	0.008	−0.056	−0.092	−0.085	0.289	−0.070	−0.184	−0.074	−0.004
	*p*	0.544	0.890	0.362	0.159	0.165	<0.01	0.281	<0.01	0.225	0.951
	n	271	269	270	235	269	269	239	270	270	270
SCC (×10^3^/mL)	r	0.110	−0.083	0.105	0.063	0.000	−0.161	−0.061	0.185	0.139	0.025
	*p*	0.070	0.174	0.083	0.332	0.995	<0.01	0.348	<0.01	0.022	0.681
	n	272	270	271	236	270	270	239	271	271	271
F (%)	r	0.057	0.077	0.043	−0.009	0.178	−0.162	0.141	0.051	−0.098	−0.099
	*p*	0.358	0.215	0.489	0.894	<0.01	<0.01	0.033	0.409	0.113	0.111
	n	261	260	261	226	260	260	231	260	260	260
P (%)	r	−0.055	−0.068	0.011	0.323	0.134	−0.292	−0.058	0.299	−0.024	0.149
	*p*	0.369	0.268	0.855	<0.01	0.028	<0.01	0.375	<0.01	0.702	0.015
	n	269	267	268	233	267	267	238	268	268	268
L (%)	r	−0.061	0.028	−0.068	−0.193	−0.072	0.227	0.066	−0.218	−0.122	0.030
	*p*	0.321	0.647	0.270	<0.01	0.245	<0.01	0.309	<0.01	0.047	0.623
	n	267	265	266	231	265	265	236	266	266	266

F—fat content in milk, P—protein content in milk, L—lactose content in milk, TA—titratable acidity, SCC—somatic cells count, r—correlation coefficient, *p*—statistical significance, n—number of observations.

**Table 4:** Influence of selected effects on milk quality and mastitis occurrence after calving.

Effect	Level	Cultured Microbial Occurrence After Calving (%)	Occurrence of Mastitis in Udder Quarters After Calving (%)	SCC After Calving (×10^3^/mL)
		LSM ± SELSM	LSM ± SELSM	LSM ± SELSM
farm	1	26.81 ± 3.071 ^A^	13.45 ± 4.306	208.98 ± 143.543
	2	8.15 ± 1.755 ^B^	12.50 ± 2.287	284.85 ± 75.608
recommendation for dry cow therapy	ATB	17.83 ± 2.378	17.19 ± 3.190 ^a^	183.63 ± 105.636
	NATB	17.13 ± 2.444	8.76 ± 3.336 ^b^	310.21 ± 110.309

SCC—somatic cells count; ATB—antibiotic treatment; NATB—non-antibiotic treatment; A, B—statistical significance … *p* < 0.01; a, b—statistical significance … *p* < 0.05.

## Data Availability

The original contributions presented in the study are included in the article. Further inquiries can be directed to the corresponding author.

## Abbreviations

The following abbreviations are used in this manuscript:

SDCT	Selective Dry Cow Therapy
SCC	Somatic Cell Count
IMI	Intramammary infections
AMR	Antimicrobial resistance
BDCT	Blanket Dry Cow Therapy
TS	Total Solids
SNF	Solids-Non-Fat
FP	Freezing Point
ATB	Antibiotic
NATB	Non-Antibiotic
MPC	Milk Performance Control
BR	Breeding records
